# A Disintegrin and Metalloproteinase (ADAM) Family—Novel Biomarkers of Selected Gastrointestinal (GI) Malignancies?

**DOI:** 10.3390/cancers14092307

**Published:** 2022-05-06

**Authors:** Marta Łukaszewicz-Zając, Sara Pączek, Barbara Mroczko

**Affiliations:** 1 Department of Biochemical Diagnostics, Medical University of Bialystok, 15-269 Bialystok, Poland; barbara.mroczko@umb.edu.pl; 2 Department of Biochemical Diagnostics, University Hospital of Bialystok, 15-269 Bialystok, Poland; sara.paczek@umb.edu.pl; 3 Department of Neurodegeneration Diagnostics, Medical University, 15-269 Bialystok, Poland

**Keywords:** ADAM, biomarker, gastrointestinal tumours

## Abstract

**Simple Summary:**

A disintegrin and metalloproteinase (ADAM) proteins are proteolytic enzymes that are responsible for destroying the extracellular matrix, but they also have adhesive properties. Recent investigations have demonstrated that the expression of several ADAMs is upregulated in gastrointestinal (GI) tumour cells and have linked the secretion of these proteins to pathogenesis of GI malignancies. Therefore, the aim of this review is to establish the involvement of selected ADAMs in the progression of GI malignancies as well as their prognostic significance. It was found that selected ADAMs might stimulate the proliferation and invasion of malignant cells and may be associated with unfavourable survival of patients with GI tumours. In conclusion, this review confirms the significance of selected ADAMs in the pathogenesis of the most common GI cancers and indicates their promising significance as potential prognostic biomarkers as well as therapeutic targets for GI malignancies.

**Abstract:**

The global burden of gastrointestinal (GI) cancers is expected to increase. Therefore, it is vital that novel biomarkers useful for the early diagnosis of these malignancies are established. A growing body of data has linked secretion of proteolytic enzymes, such as metalloproteinases (MMPs), which destroy the extracellular matrix, to pathogenesis of GI tumours. A disintegrin and metalloproteinase (ADAM) proteins belong to the MMP family but have been proven to be unique due to both proteolytic and adhesive properties. Recent investigations have demonstrated that the expression of several ADAMs is upregulated in GI cancer cells. Thus, the objective of this review is to present current findings concerning the role of ADAMs in the pathogenesis of GI cancers, particularly their involvement in the development and progression of colorectal, pancreatic and gastric cancer. Furthermore, the prognostic significance of selected ADAMs in patients with GI tumours is also presented. It has been proven that ADAM8, 9, 10, 12, 15, 17 and 28 might stimulate the proliferation and invasion of GI malignancies and may be associated with unfavourable survival. In conclusion, this review confirms the role of selected ADAMs in the pathogenesis of the most common GI cancers and indicates their promising significance as potential prognostic biomarkers as well as therapeutic targets for GI malignancies. However, due to their non-specific nature, future research on ADAM biology should be performed to elucidate new strategies for the diagnosis of these common and deadly malignancies and treatment of patients with these diseases.

## 1. Gastrointestinal Cancers—General Characteristics

According to estimates from the American Cancer Society, in 2018 there were approximately 1.7 million new cancer cases and 610,000 cancer deaths [1]. Gastrointestinal (GI) cancers are defined as a group of malignancies that includes cancers of the liver, oesophagus, gallbladder, pancreas, stomach, large and small intestine and anus [2,3,4]. Neoplasms originating from the GI tract, including colorectal cancer (CRC) and gastric cancer (GC), are among the five most common malignancies in both men and women worldwide. It is estimated that CRC is one of the most frequently diagnosed cancers and the second most common cause of cancer-related deaths worldwide. Gastric cancer is the fourth most common cancer and accounts for 8.8% of all cancer-related deaths [2,3,4].

The incidence of GI cancers shows significant geographical variation. CRC incidence is higher in Western Europe and North America, while the incidence of GC and liver cancer (LC) is elevated in Asia and Africa. The main risk factors for GI cancers include tobacco and alcohol intake, genetic factors, viruses such as Papillomaviruses, Epstein–Barr virus (EBV) or hepatitis B and C. Other risk indicators that may cause tumour development are bacterial infections and microbiome imbalance, *Helicobacter pylori* infection, as well as an unhealthy diet and obesity. It was suggested that *Fusobacterium nucleatum* plays a role in CRC development via promotion of tumour progression include generating a proinflammatory tumour-promoting microenvironment [5,6,7]. Moreover, the study of Yoshimura revealed that *Helicobacter pylori* stimulated temporal changes in the levels of proteolytic enzymes such as ADAM10 and ADAM17 transcripts in gastric epithelial cells, while chronic infection with *Helicobacter pylori* may result in persistent mucosal increases in members of the ADAM family [8]. Some epidemiological studies have shown an increased risk of GI cancers in overweight and obese individuals, while substantial evidence has linked reduced physical activity with an increased risk of colon cancer. Moreover, it has been proven that a high salt intake is associated with enhanced prevalence of GC, whereas a diet high in red and processed meats has been linked to an increased risk of GC, EC, PC and CRC [2,3,4,5,6,7,8,9]. Furthermore, changes in lifestyle, the growing population and environmental factors as well as advances in medicine may also affect the epidemiology of GI cancers [2].

Clinical symptoms of GI cancers depend on the type of malignancy and tumour stage as well as the development of systemic symptoms such as early satiety nausea, anorexia, changes in the sense of smell, stress or dysgeusia [10]. It has been revealed that fatigue is a major sign followed by pain, anxiety, poor well-being, sleep disturbances, poor appetite, depression, drowsiness, dyspnoea and nausea [10,11]. Moreover, in EC, GC and PC, signs of disease may occur early. However, symptoms are directly related to the cancer or release of inflammatory cytokines [10,11]. Neoplasms of the alimentary tract are characterized by rapid progression and a very unfavourable prognosis. The diagnostic process of GI cancers includes endoscopic evaluation as an important tool in diagnosis and staging. Diagnosis is confirmed with an upper gastrointestinal endoscopy and biopsy. Other imaging tests useful in diagnosis of GI tumours include computed tomography (CT), positron emission tomography–CT (PET–CT) and endoscopic ultrasound (EUS) [12]. It is also recommended that apart from imaging tests, diagnosis of GI tumours should include laboratory tests. Measurements of the well-investigated classical tumour markers for GI malignancies such as carcinoembryonic antigen (CEA), cancer antigen 19-9 (CA 19.9), cancer antigen 50 (CA 50) or cancer antigen 72.4 (CA 72.4) are not useful in the early detection of these malignancies due to their low diagnostic sensitivity and specificity. A number of candidates for novel biochemical markers for GI malignancies such as matrix metalloproteinases and their tissue inhibitors, cytokines and chemokines as well as specific proteins such as C-reactive protein or interleukin-6 have been evaluated by scientists in the last decade. However, no studies have confirmed their significance in early diagnosis of GI malignancies [13,14,15,16,17,18,19,20,21,22,23]. Therefore, future research should focus on the search for new, non-invasive and easily accessible biomarkers characterized by high diagnostic sensitivity and specificity, which would be useful in the early detection and tumour staging as well as improve treatment implementation.

Remodelling of the extracellular matrix (ECM) plays an important role in tumour progression, including growth, proliferation and angiogenesis. Moreover, many authors link these proteases to tumour invasiveness, particularly metastasis [13]. Therefore, in our previous studies we assessed the usefulness of selected MMPs and their tissue inhibitors in the diagnosis and progression of GI malignancies such as CRC, PC, EC and GC. In addition, selected MMPs, such as MMP-14, are able to regulate a variety of signalling pathways and cell functions, including apoptosis. It has been proven that the proteolytic activity of MMPs is physiologically inhibited by tissue inhibitors of metalloproteinases (TIMPs). TIMP-2 regulates several cell functions including migration, proliferation and apoptosis through MMP-dependent and -independent mechanisms, e.g., via inhibition of FGF-2-induced endothelial cell proliferation, suppression of the mitogenic activity of epidermal growth factor (EGF) or inhibition of angiogenic factor-induced endothelial cell proliferation and angiogenesis [24]. Our previous results indicated that selected MMPs, especially MMP9, might be used as potential biomarkers in the diagnosis and progression of several GI malignancies [14,15,16,17,18,19]. Based on the most recent literature reports, we believe that another member of the MMP family—ADAM (A disintegrin and metalloproteinase)—might be considered a potential candidate for a biochemical marker involved in GI carcinogenesis because of both proteolytic and adhesive activities. Thus, the goal of our review is to summarize the current knowledge concerning the significance of selected ADAMs in the pathogenesis of GI cancers and their potential utility in diagnosis and prognosis of patients with these malignancies.

## 2. A Disintegrin and Metalloproteinase (ADAM)—General Information

A disintegrin and metalloproteinases (ADAMs) belong to the family of zinc-dependent proteases, such as metalloproteinases, which consist of 21 members, 13 of which have proteolytic activity [25,26]. They are also known as metalloproteinase, disintegrin, cysteine-rich (MDC) proteins. ADAMs regulate the shedding of membrane-bound proteins, cytokines, growth factors as well as ligands and receptors. It has been demonstrated that the structure of most of these proteins consists of a prodomain, a metalloprotease region, a disintegrin domain for adhesion, a cysteine-rich region, epidermal-growth-factor (EGF) repeats, a transmembrane module as well as a cytoplasmic tail [27,28].

**Figure 1:**
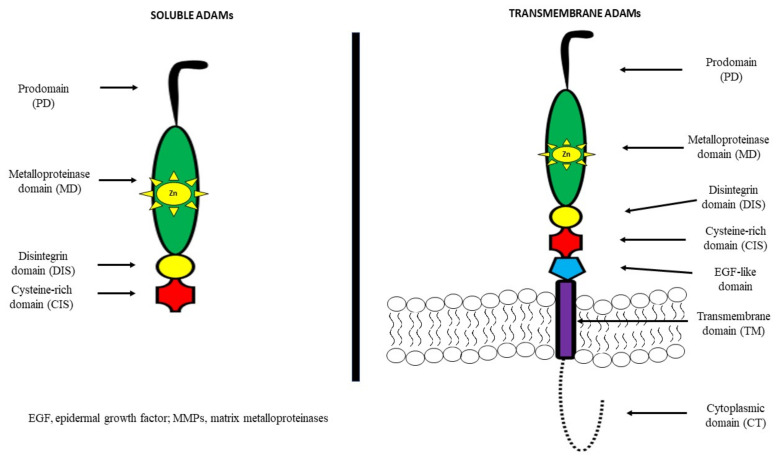
Structure of ADAMs of transmembrane and soluble ADAMs [30,31,32].

It has been observed that among other cell-surface proteins, ADAMs are unique because of both adhesive and proteolytic activities. Moreover, it has been indicated that EGF repeats and the cysteine-rich region mediate cell fusion or the interaction of these proteins with other molecules [29,30,31,32]. ADAMs are mostly transmembrane proteins, but selected ADAMs, such as ADAM11, 12, 17 and 28 may generate a soluble, secreted protein. About 50% of the ADAM family consists of a metalloproteinase domain with a catalytic site consensus sequence that allows for protein–protein interactions [27,28]. The structures of both transmembrane and soluble ADAMs are presented in Figure 1 [30,31,32].

Several ADAM genes initiate more than one protein due to differential splicing of mRNA, a post-transcriptional modification in which a single gene can code for multiple proteins. This promotes the synthesis of a secreted ADAM structure, in addition to membrane-anchored forms, or variation in the length of the cytoplasmic tail of ADAM proteins. According to differences in the active site sequence of the metalloproteinase domain, 60% of the members are non-proteolytic ADAM molecules. By contrast, active sites in the metalloproteinase domain of the proteinase-type ADAM molecules (ADAM8, 9, 10, 12, 15, 17, 19–21, 28, 30 and 33) contain a common HEXGHXXGXXHD sequence with a ‘Met-turn’ which is also present in the catalytic metalloproteinase domain of MMP members [33]. ADAM10 and ADAM17 have a similar structure and are characterized by a membrane-proximal domain in the extracellular region in place of the EGF-like, cysteine-rich domain that provides substrate recognition [34,35]. A distinct subfamily of ADAMs is a disintegrin and metalloproteinase with thrombospondin motifs (ADAMTS), which consists of a pro-domain and a disintegrin, metalloproteinase, and cysteine-rich domain. In addition, ADAMTS have a specific thrombospondin motif instead of a transmembrane domain [27,28,29].

Growing evidence indicates that ADAM proteases are expressed in an inactive form. It has been proven that the activity of ADAMs and ADAMTS is regulated via endogenous inhibitors called tissue inhibitors of metalloproteinases (TIMPs) as well as proprotein convertases. In addition, they might also be controlled by protein kinase C activators, G-protein coupled receptor agonists or Ca2+ ionophores. Various ADAMs are usually kept in an inactive state because of the interaction of a cysteine residue at the propeptide domain with zinc in the metalloproteinase module. Therefore, ADAMs and MMPs are activated by the cysteine switch mechanism that disrupts the cysteine–zinc interaction to expose the catalytic site. It has been observed that levels of these proteins are regulated at the transcriptional level. However, current knowledge concerning the role of physiological inhibitors of ADAMs is still limited. It has been proven that only TIMP3 inhibits the crystal structure of the protease domain of human ADAM17, while ADAM10 might be inhibited by TIMP1 and 3, and by hydroxamates [35]. TIMP3 may be an inhibitor of various ADAMs as well as ADAMTS members [27,28,29]. Increasing numbers of reports have demonstrated that GI cancer cells might be transformed by aberrant and uncontrolled mechanisms that may produce alternative splicing. It was found that the *APC* gene, aberrant splice skipping of exon 4, as well as *Ron* gen, skipping of exon 11 are involved in colon cancer progression. An alternative 5’ splice site in BCL-X, involved in apoptosis, is overexpressed in hepatocellular carcinoma, similar to the *CDH17* gene (exclusion of exon 13) that regulates incidence of tumour recurrence. In addition, the *TACC1* gene has splicing variants associated with GC. These findings suggest the expression of aberrant and abnormal splice variants in GI cancer development [36,37,38,39].

ADAMs, similarly to MMPs, possess various physiological functions and the ability to regulate many processes such as cell migration, proliferation, angiogenesis, apoptosis, wound healing, tissue repair and survival. By way of illustration, ADAM1 and 2 are able to modulate cell adhesion or sperm–egg fusion. In addition, ADAM12 plays a role in myoblast fusion, while ADAM9, 10 and 17 regulate ectodomain shedding of cell-surface proteins [27,28,29]. However, these molecules might also be involved in some pathological conditions such as cardiovascular and malignant diseases, including GI cancers [40,41,42,43].

## 3. A Disintegrin and Metalloproteinase (ADAM)—Their Role in Tumour Development

A growing body of evidence suggests that cancer growth is not only driven by tumour cell-intrinsic mechanisms, but might be dependent on paracrine signals, such as growth factors or cytokines, produced by the tumour microenvironment (TME). These molecules are synthesized as trans-membrane proteins and must be released by limited proteolysis defined as ectodomain shedding. It has been proven that ADAMs are major mediators of ectodomain shedding and thus are able to initiate paracrine signal transduction [25]. Changes in the activation process of paracrine signal transduction is a crucial step in the development of GI malignancies. Therefore, ADAM proteases play an important role in inflammation as well as pathogenesis of several tumours because of their ability to interact with a variety of substrates [25].

The multiple functional roles of ADAMs prove their involvement in a variety of normal and pathophysiological conditions, including cancer progression [32,41,42,44]. Characteristics of selected ADAMs [32] and their significance in tumour biology are presented in Table 1.

It has been reported that ADAM8 is involved in tumour cell migration and invasion, ADAM9 plays a role in tumorigenesis, invasion and metastasis through modulation of growth factor activity and integrin function, while overexpression of ADAM10 appears to promote the growth and proliferation of tumour cells. In addition, ADAM12 cleaves various ECM molecules including gelatin, type IV collagen and fibronectin, suggesting a potential role of this enzyme in ECM digestion in cancer invasion and metastasis. Thus, ADAM12 functions as a shedder, adhesion molecule and ECM-degrading proteinase and is involved in cancer progression. ADAM28 may play a key role in cancer cell proliferation and metastasis, whereas ADAM17 is a target of tumorigenesis, but the role of ADAM15 in cancer biology remains to be elucidated [32,44].

**Figure 2:**
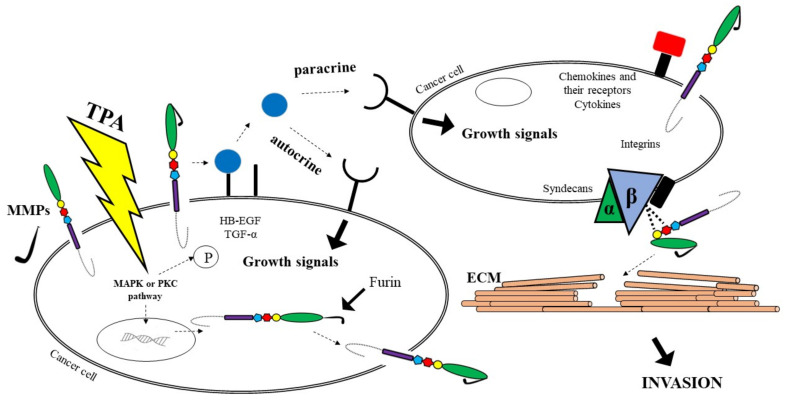
The significance of ADAMs in cancer biology [30,31,32].

It has been established that ADAM12 and ADAM28 regulate the level of free IGF-1 by proteolysis of the IGFBP-3/IGF-1 protein complex [44]. In addition, ADAM17 protein is responsible for the activation of TNFα, initiating the signalling pathway associated with the EGF receptor for which it is a ligand, leading to tumour cell proliferation [45]. Adamalysines might be involved in the pathogenesis of gastric cancer via the EGFR signalling pathway and the TGF-α/Smad pathway [46]. In vitro assays indicate that ADAM8 overexpression promotes cell growth and increases migration and invasion abilities by decreasing the p-p38/p-extracellular regulated protein kinase (p-ERK) ratio. Several studies suggest that there are five most common pathways of ADAMs involvement in cancer biology. It has been reported that proADAM might be activated via furin or MMPs. As furin activates MMP activity, cancer cells have the potential to become metastatic. Another pathway leads to growth factors such as TGFα shedding, which may change signals on the cancer cell’s surface. Soluble growth factors activate EGFR on cells, causing the enhanced cells proliferation via autocrine and paracrine manners. A third route involves the participation of ADAMs as adhesion molecules with integrins on cells, which may facilitate the digestion of the substrates of the ECM. Fourth, cell proliferation signals can be regulated indirectly by ADAMs via integrins; thus these molecules provide traction to migrating cells through the ECM using integrins. In addition, ADAMs are able to stimulate cancer development and metastasis via the interaction with other molecules, including cytokines and their receptors, that are also associated with cancer progression. ADAMs, similar to MMPs, are able to cleave ECM molecules. As a consequence, it allows neoplastic cells to adhere to new locations, which is responsible for, e.g., cancer metastasis [44]. All the pathways are presented in Figure 2.

Recent studies indicate the importance of the ADAM family in tumour formation, migration, proliferation and development [47,48]. There is increasing evidence that several ADAMs are differentially expressed in tumours. Some studies have confirmed the role of ADAMs in the biology of malignant cells, including breast [49,50], renal [51] and small cell lung [52] cancer as well as GI malignancies such as gastric [46,47,48,49,50,51,52,53,54,55,56,57,58], colorectal [59,60,61,62] and pancreatic [63,64,65] cancer and hepatocellular carcinoma [66,67,68,69].

## 4. A Disintegrin and Metalloproteinase (ADAMs)—Their Role in the Development and Prognosis of Gastrointestinal Cancers (GI)

Mounting evidence has associated an increased expression of individual ADAM family members with various types of cancer [46]. Among ADAM proteins, proteinase activities have been demonstrated for ADAM8, 9, 10, 12, 15, 17, 19, 28 and 33 [32]. Therefore, this review will focus of their significance in the pathogenesis of GI cancers. The significance of selected ADAMs in GI malignancies is presented in Table 2.

## 5. A Disintegrin and Metalloproteinase 8 (ADAM8)

Several studies have demonstrated that adamalysines are highly expressed in gastric cancer and play an important role in gastric cancer proliferation and invasion [46,55,56]. A disintegrin and metalloprotease 8 (ADAM8) is a member of the ADAM family which is involved in tumour development by enhancing cellular abilities of invasion and migration [60,64,73], stimulating angiogenesis [73,74] and inhibiting cancer cell apoptosis [75]. In GI malignancies, overexpression of this protease has been reported in pancreatic [64], gastric, colorectal and hepatocellular carcinomas [68].

ADAM8 plays an important role in GC proliferation and invasion [54]. A study by Huang et al. evaluated the clinical significance of ADAM8 in GC and explored its biological effects on GC. Using quantitative reverse transcription-polymerase chain reaction (q-RT-PCR), Western blotting and immunohistochemical (IHC) staining analysis, the authors revealed that ADAM8 mRNA expression was significantly upregulated in GC tissues compared with noncancerous tissues. Positive ADAM8 expression was more frequent in GC tissues in comparison to normal tissues and correlated with tumour size (T factor), N (nodal involvement), vessel invasion as well as shorter GC patient overall survival [54]. In addition, ADAM8 overexpression promoted cell growth and increased their migration and invasion abilities. The authors concluded that ADAM8 is able to promote proliferation and invasion of GC cells, and its expression is positively correlated with poor survival. Therefore, this glycoprotein might be a promising target in GC therapy [54].

Yang et al. assessed the expression of ADAM8 in CRC using q-RT-PCR Western blot and IHC staining analysis [60]. Expression of mRNA and protein levels of ADAM8 were significantly elevated in CRC tissues in comparison to adjacent normal tissues, which may suggest its importance in CRC carcinogenesis [47]. In addition, knockdown of ADAM8 in two CRC cell lines stimulated apoptosis and reduction of cellular growth and proliferation [60]. There was no significant association between ADAM8 expression and the clinicopathological characteristics of the tumour, which was confirmed using the IHC method [60]. Moreover, survival analysis indicated that CRC patients with ADAM8-positive tumours had worse 5-year overall survival and 5-year disease free survival in comparison with patients with ADAM8-negative tumours. Additionally, multivariate analysis revealed that ADAM8 expression was an independent prognostic factor for the survival of CRC patients [60]. The authors concluded that ADAM8 is overexpressed in CRC and may promote tumour growth as well as serve as an independent biomarker for the survival of CRC patients [60].

A study by Valkovskaya et al. [64] demonstrated that ADAM8 mRNA was significantly overexpressed in PC compared to normal pancreatic tissues, while elevated ADAM8 mRNA and protein expression levels correlated with reduced survival among PC patients. In addition, silencing of ADAM8 expression did not have a significant impact on PC cell growth but was able to suppress the invasiveness of this malignancy. The authors concluded that ADAM8 is overexpressed in PC tissue and may promote cancer cell invasiveness as well as correlate with reduced PC survival [64].

It has been reported that ADAM8 expression is markedly elevated in hepatocellular carcinoma (HCC) tissues in comparison to normal liver tissues. In addition, enhanced expression of this protein is positively correlated with elevated concentrations of the classical tumour marker for HCC—alpha-fetoprotein (AFP), tumour stage and size, histological differentiation, tumour recurrence and tumour metastasis [65]. Moreover, significantly shorter overall survival rates are observed among HCC patients with elevated ADAM8 expression in comparison to patients with low expression of this glycoprotein. Furthermore, multivariate analysis suggests that ADAM8 expression might be an independent prognostic factor for the survival of patients with HCC [65].

## 6. A Disintegrin and Metalloproteinase 9 (ADAM9)

ADAM metalloproteinase domain-containing protein (ADAM9) has been reported to be overexpressed in several GI cancers. Using immunohistochemistry and q-RT-PCR, it was demonstrated that this molecule was significantly upregulated in GC compared to non-neoplastic foveolar epithelium [46]. The administration of anti-ADAM9 antibodies inhibited the development of this malignancy, while ADAM9 promoted malignant growth of GC cells. The authors suggest that ADAM9 influences tumour cells via two possible ways—interaction with adhesion molecules, or the proteolytic ‘shedding’ of signalling molecules, which leads to the activation of their receptors, including the EGF receptor and its ligands [46]. These investigations indicate that modulation of the tumour–host interface may contribute to the pathogenesis, development and progression of GC [46].

It has also been reported that ADAM9 is overexpressed in PC and its cell lines, which has been proven using gene expression profiling by microarray. Moreover, based on the IHC method, the authors report that ADAM9 expression is associated with poor tumour differentiation and a worse prognosis of PC patients [65,70]. In addition, a study by Yamada et al. [63] revealed that PC cells expressed significantly higher levels of ADAM9 in comparison to normal pancreatic epithelial cells, which may suggest that this protein plays a role in the progression of PC and may present promising target for the diagnosis of this malignancy and treatment of patients [63].

## 7. A Disintegrin and Metalloproteinase 10 (ADAM10)

The metalloproteinase domain-containing protein 10 (ADAM10) has been implicated in the development and progression of several GI malignancies, including GC, HCC and CRC [56].

Protein levels of ADAM10 were upregulated in GC tissues compared with adjacent non-cancerous tissues, which was assessed using the IHC method. In addition, positive ADAM10 expression correlated with TNM stage, size and location of the tumour, depth of invasion as well as lymph node and distant metastases. The 5-year survival rate for patients with stage I, II and III GC with high expression of ADAM10 was significantly lower than for patients with low immunoreactivity of this protein. In addition, multivariate analysis determined that upregulation of ADAM10 was an independent prognostic indicator of GC [56]. The authors concluded that the expression of this protein is significantly associated with the presence of lymph node and distant metastases and poor prognosis and proved that ADAM10 could be a predictor of tumour progression and prognosis [56].

A study by Walkiewicz et al. [61] evaluated the concentrations of serum ADAM10 in CRC patients using the ELISA method. The authors revealed that serum ADAM10 levels were significantly higher in CRC patients in comparison to healthy controls and correlated with the clinical stage of CRC. In addition, there was a relationship between ADAM10 concentrations and the histological grade of the tumour, as serum ADAM10 levels were elevated in G1 tumours when compared to G3 malignancies. In conclusion, this protein might be a predictor of tumour progression [61].

Some clinical investigations have reported ADAM10 overexpression in HCC, which correlated with the presence of metastasis, grade, differentiation and size of the tumour. Moreover, the authors indicated that ADAM10 protein expression was significantly associated with reduced patient survival and may serve as a useful molecular marker for HCC [69]. Elevated ADAM10 expression has also been found in HCC cells, which correlated with the increased capacity of HCC cells for proliferation, invasion and migration, suggesting that this protein plays an important role in HCC progression [66,67].

## 8. A Disintegrin and Metalloproteinase 12 (ADAM12)

ADAM12 is a proteolytic glycoprotein that is located almost exclusively in tumour cells [61]. Moreover, tumour-associated stroma may also stimulate the expression of this protein in tumour cells via the synthesis of tumour growth factor β1 (TGF β1) that promotes carcinogenesis [76]. Some clinical investigations have revealed that ADAM12 overexpression may lead to increased tumour size and metastasis. Two isoforms of this glycoprotein have been discovered. However, only the secreted form of ADAM12 may enhance the ability of tumour cells to migrate and invade as well as stimulate local and distant metastasis in vivo. Moreover, it has been indicated that the stimulatory effect of ADAM12 on the migration and invasion of tumour cells is probably dependent on its proteolytic activity, and thus ADAM12 may represent a potential therapeutic target [30,36]. Using IHC and q-RT-PCR, the transcription and expression pattern of ADAM12 in GC cells and the corresponding non-tumour tissue as well as in GC cell lines were examined. Furthermore, immunoreactivity of this glycoprotein was significantly upregulated in GC compared to non-neoplastic foveolar epithelium [46]. Moreover, ADAM-specific antibodies enhanced the proliferation of GC cell lines. The authors concluded that this protein is implicated in the malignant growth of GC cells due to the interaction with adhesion molecules, the proteolytic ‘shedding’ of signalling molecules and the activation of their receptors, including the epithelial growth factor (EGF) receptor and its ligands [46].

Serum ADAM12 levels in patients with CRC have also been evaluated using the immunoenzyme method [43,61]. Concentrations of this glycoprotein were higher in the sera of CRC patients in comparison to healthy controls, while the highest concentrations were found in advanced stages of CRC, which may suggest the significance of ADAM12 in the pathogenesis of this malignancy [43,61].

## 9. A Disintegrin and Metalloproteinase 15 (ADAM15)

ADAM15 as a membrane protein with an adhesion domain is able to bind to a5b1 integrin via a unique arginine–glycine–aspartic acid (RGD) motif domain.

ADAM15 was found to be significantly upregulated in GC compared to non-neoplastic foveolar epithelium, with the expression being higher in intestinal neoplasms than in diffuse-type tumours, which was assessed using IHC and q-RT-PCR analyses [46]. The authors also demonstrated that the anti-ADAM15 antibodies inhibited GC cell growth, while the protein was also implicated in the malignant growth of GC cells, similarly to ADAM12, via the interaction with proteolytic ‘shedding’ of signalling molecules or adhesion molecules [46].

It has been shown that 63% of CRC cells demonstrate reduced expression of ADAM15 in cancer cells, which has been evaluated at the mRNA level. Moreover, downregulation of this molecule is associated with histologically poorly differentiated malignancies. Toquet et al. [62] assessed ADAM15 expression in colon carcinomas using both IHC and mRNA quantitative methods [49]. The authors observed decreased expression of ADAM15 in CRC associated with a loss of differentiation in a subset of colon carcinomas, which indicates that the role of ADAM15 in cancer progression is tissue-specific [62].

Some clinical investigations have revealed that the mRNA expression of ADAM15 is significantly higher in PC cells than in normal pancreatic epithelial cells, which may indicate the involvement of ADAM15 in the development of this malignancy [63].

## 10. A Disintegrin and Metalloproteinase 17 (ADAM17)

A disintegrin and metalloproteinase 17 (ADAM17) is also known as tumour necrosis factor-alpha (TNFα) converting enzyme (TACE). A growing body of evidence has revealed that this protein is associated with inflammation and cancer [53,77,78,79]. In addition, the significance of ADAM17 in cancer development is based on its direct effect on the release of TNFα [43,45]. Therefore, the relationship between ADAM17 levels and GI malignancies has been described in several studies [71,72,80,81,82].

The overexpression of ADAM17 may enhance the migratory ability of GC cells and tumour growth [72,80]. The authors demonstrated that ADAM17 overexpression was associated with an advanced TNM stage and presence of lymph node metastasis, while there was no significant correlation between ADAM17 expression and tumour differentiation [71,72,80,81,82]. In addition, elevated ADAM17 levels correlated with reduced overall survival rates, and thus ADAM17 was found to be a significant biomarker for poor prognosis in GC [71,72,80,81,82] and shown to play an important role in the development and progression of this malignancy [81]. Similar results were presented in a study by Ni et al. who evaluated the prognostic significance of ADAM17 and its association with the clinicopathological characteristics of GC [53]. The meta-analysis revealed that lower ADAM17 levels were correlated with longer overall survival rates in GC, while ADAM17 overexpression was associated with an advanced TNM stage and the presence of lymph node metastasis. The authors concluded that ADAM17 is a significant prognostic factor for GC [53].

It has been reported that ADAM17 may enhance the malignant potential of CRC cells via increasing their motility and the expression of proangiogenic factors, promoting tumour progression and metastasis [43,61]. A study by Walkiewicz et al., based on the ELISA method, revealed a statistically significant relationship between serum levels of ADAM17 and the clinical stage of CRC. The concentrations of this protein were lower in the sera of CRC patients in comparison to the control group [43,61]. Moreover, elevated serum concentrations of ADAM17 were found in obese CRC patients, which may explain the relationship between a Western diet and the activation of malignant processes [43,61]. Other investigations have confirmed the crucial role of ADAM17 in the pathogenesis of CRC and revealed that the use of a specific anti-ADAM17 antibody may inhibit the growth of CRC cell lines [43,59,61].

## 11. A Disintegrin and Metalloproteinase 28 (ADAM28)

Disintegrin and metalloproteinase 28 (ADAM28) is also associated with the growth and metastasis of various GI malignancies. ADAM28 protease supports cancer cell proliferation, survival and migration as well as metastatic progression [83]. A study by Yin et al. [58], which used, i.a., Western blot, q-PCR, wound healing assay and flow cytometry, demonstrated ADAM28 overexpression in GC cells, which correlated with shorter overall survival in comparison to those with low ADAM28 expression [58]. In addition, the authors revealed that ADAM28 from the endothelium and GC may cleave von Willebrand Factor (WF) to eliminate vWF-induced apoptosis of GC cells and promote a pro-metastasis effect [58]. These findings indicate that ADAM28 is able to regulate GC cell proliferation, migration and apoptosis.

The significance of ADAM28 in CRC pathogenesis has also been studied. Serum levels of ADAM28 were shown to be significantly higher in CRC patients than in healthy controls. Moreover, there was a relationship between serum levels of ADAM28 and clinical TNM stage as well as the presence of distant metastases (M factor). ADAM28 concentrations were highest in patients with stage IV CRC and presence of distant metastasis. In addition, there was a significant relationship between serum ADAM28 levels and histopathological grading (G factor). Serum concentrations of this molecule were higher in G3 patients in comparison to G1 and G2 subjects [61]. The authors indicated the potential usefulness of this molecule in the pathogenesis of CRC.

## 12. Conclusions

GI cancers are among the five most common malignancies in both men and women worldwide. Therefore, there is an urgent need for more research on early biomarkers of these malignancies. Pro-tumour functions have been mostly related to proteolytic enzymes from the metalloproteinase family including A disintegrin and metalloproteinases (ADAMs). Some clinical investigations suggest the potential role of these proteins in the pathogenesis of GI cancers. In this paper, we reviewed the involvement of selected ADAMs in GI cancer development and progression. The present paper demonstrates that of all ADAMs, ADAM8 is able to promote progression of CRC, PC, GC and HCC cells and may serve as a prognostic factor. ADAM9 contributes to the pathogenesis of GC and PC and poor PC patient prognosis. ADAM10 is significantly associated with the presence of lymph node and distant metastases in GC and histological grading in CRC patients. In addition, elevated ADAM10 levels correlate with a worse prognosis of GC and HCC patients. ADAM12 and ADAM15 are implicated in the malignant growth of GC, CRC and CRC. ADAM17 is associated with an advanced TNM stage and the presence of lymph node metastasis and is a significant biomarker for poor prognosis in GC. It might also play a role in the pathogenesis of CRC. ADAM28 levels correlate with the TNM stage, the presence of distant metastasis and the histological grading of CRC and may produce a pro-metastasis effect on GC.

In conclusion, our review paper confirms that selected ADAMs, particularly those with proteolytic properties, play an important role in the development of GI tumours and patient prognosis. However, given the non-specific nature of adamalysines, further research needs to be performed before selected ADAMs can be established as biomarkers for GI malignancies.

## Figures and Tables

**Table 1:** Characteristics of selected ADAMs [32].

ADAMs	Other Name	Involvement in Cancer Biology	Inhibitors
ADAM8	MS2 (CD156)	Promotion of migration	-
ADAM9	MDC9, MCMP, Meltrin-γ	Promotion of cell adhesion and invasion, binding to integrins (α6β4 and α2β1)	-
ADAM10	MDAM, Kuzbanian	Type I membrane glycoprotein L1 shedding, promotion of cell growth and migration	TIMP1 TIMP3
ADAM12	Meltrin-α, MCMP, MLTN, MLTNA	HB-EGF (heparin-binding epidermal growth factor) shedding, promotion of cell growth	TIMP3
ADAM15	Metargidin, MDC15, AD56, CR II-7	Promotion of cell growth	No data
ADAM17	TACE, cSVP	TGF-β (transforming growth factor) shedding, promotion of cell growth	TIMP2 TIMP3
ADAM19	Meltrin-β, FKSG34	No data	-
ADAM28	e-MDC II, MDC-Lm, MDC-Ls	IGFBP-3 (insulin-like growth factor binding protein-3) cleavage, promotion of cell growth	TIMP3 TIMP4
ADAMTS1	C3-C5, METH1, KIAA1346	HB-EGF (heparin-binding epidermal growth factor) and AR shedding, promotion of cell growth, survival and invasion	No data
ADAMTS4	KIAA0688, aggrecanase-1, ADMP-1	No data	TIMP3
ADAMTS5	ADAMTS11, aggrecanase-2, ADMP-2	Brevican cleavage, promotion of invasion	TIMP3

**Table 2:** The significance of selected ADAMs in GI malignancies.

ADAMs	GI Cancers	Results	References
ADAM8	GC	overexpression in GC tissues compared with noncancerous tissues correlation with tumour size (T factor), N (nodal involvement), vessel invasion and shorter survival	[54]
	CRC	overexpression in CRC tissues compared with adjacent normal tissues independent prognostic factor for patient survival	[60]
	PC	overexpression in PC compared with normal pancreatic tissues correlation with reduced patient survival	[64]
	LC	overexpression in HCC tissues compared with normal liver tissues correlation with higher concentrations of alpha-fetoprotein, tumour stage and size, histological differentiation, tumour recurrence and tumour metastasis independent prognostic factor for patient survival	[68]
ADAM9	GC	overexpression in GC compared with non-neoplastic foveolar epithelium	[46]
	PC	overexpression in PC cell lines compared with normal epithelial cells correlation with poor tumour differentiation and worse patient prognosis	[63,65,70]
Adam10	GC	overexpression in GC lesions compared with adjacent non-cancerous tissues correlation with TNM stage, size and location of tumour, depth of invasion, presence of lymph node and distant metastases independent prognostic indicator of GC	[56]
	CRC	elevated serum concentrations in CRC patients in comparison to healthy controls correlation with clinical stage and histological grade of tumour predictor of tumour progression	[61]
	HCC	overexpression correlated with the presence of metastasis, grade, differentiation and size of tumour correlation with reduced patient survival	[66,67,69]
ADAM12	GC	upregulated expression in GC compared with non-neoplastic foveolar epithelium	[46]
	CRC	serum levels were higher in the sera of CRC patients in comparison to healthy subjects highest concentrations found in advanced stage of CRC	[43,61]
ADAM15	GC	upregulated in GC compared with non-neoplastic foveolar epithelium implicated in malignant growth of GC cells	[46]
	CRC	reduced expression of ADAM15 in cancer cells correlation with histologically poorly differentiated malignancies	[62]
	PC	overexpression in PC cells compared with normal pancreatic epithelial cells	[63]
ADAM17	GC	overexpression promoted migration of GC cells and tumour growth overexpression was associated with advanced TNM stage and presence of lymph node metastasis a significant biomarker for poor prognosis in GC	[71,72]
	CRC	decreased serum levels in CRC patients in comparison to healthy controls	[43,59,61]
ADAM28	GC	overexpression in GC cells regulated cell proliferation, migration and apoptosis	[58]
	CRC	elevated serum levels in CRC patients in comparison to healthy subjects correlation with clinical TNM stage, presence of distant metastases (M factor) and histopathological grading (G factor)	[61]
